# Selenium Content in Seafood in Japan

**DOI:** 10.3390/nu5020388

**Published:** 2013-01-31

**Authors:** Yumiko Yamashita, Michiaki Yamashita, Haruka Iida

**Affiliations:** National Research Institute of Fisheries Science, Kanazawa, Yokohama, Kanagawa, 236-8648, Japan; E-Mails: mic@affrc.go.jp (M.Y.); harukaiida@affrc.go.jp (H.I.)

**Keywords:** selenium, mercury, food safety, muscle, fish, seafood

## Abstract

Selenium is an essential micronutrient for humans, and seafood is one of the major selenium sources, as well as red meat, grains, eggs, chicken, liver and garlic. A substantial proportion of the total amount of selenium is present as selenium containing imidazole compound, selenoneine, in the muscles of ocean fish. In order to characterize the selenium content in seafood, the total selenium levels were measured in the edible portions of commercially important fish and shellfish species. Among the tested edible portions, alfonsino muscle had the highest selenium levels (concentration of 1.27 mg/kg tissue). High levels of selenium (1.20–1.07 mg/kg) were also found in the salted ovary products of mullet and Pacific herring. In other fish muscles, the selenium levels ranged between 0.12 and 0.77 mg/kg tissue. The selenium levels were closely correlated with the mercury levels in the white and red muscles in alfonsino. The selenium content in spleen, blood, hepatopancreas, heart, red muscle, white muscle, brain, ovary and testis ranged between 1.10 and 24.8 mg/kg tissue in alfonsino.

## 1. Introduction

Selenium is an essential nutrient for humans [1,2,3]. Selenium is a constituent of antioxidant enzymes and proteins, such as glutathione peroxidases, thioredoxin reductases and selenoprotein P, which contain one or more selenocysteine residues [1,2,3]. Selenium bioavailability may depend on the food source and chemical forms of selenium [4]. The major compounds in plant foods are selenite, selenomethionine and γ-glutamyl methylselenocysteine [4]. Although there are limited data on the chemical forms of organic selenium in foods of animal origin, the major chemical form of selenium is considered to be selenocysteine and selenomethionine that are incorporated in muscle proteins [4].

The dietary intake of selenium by fish consumption may be important for the enhancement of selenium redox function. Fish are estimated to be the major selenium source in Japan, contributing to approximately 35% of the total selenium consumed [1]. Therefore, the selenium content and its chemical forms in the edible portions of fish and shellfish should be characterized.

Recent studies showed that the fish muscles of tuna and other predatory fish contain high levels of the selenium-containing imidazole compound, 2-selenyl-*N*_α_, *N*_α_, *N*_α_-trimethyl-l-histidine (selenoneine) [5,6,7]. This compound was identified as the major organic selenium in the blood and muscle tissue of tuna [5,6,7]. Selenoneine contains an imidazole ring with a unique selenoketone group and has radical scavenging activity [5]. Therefore, the dietary intake of selenoneine through fish consumption is thought to be important for enhancing antioxidant effects in tissues and cells. 

Selenium intake reduces methylmercury (MeHg) toxicity [8,9,10,11]. The risk of fish consumption is thought to be provided by measuring not only the MeHg exposure, but also the levels of selenium and other components that benefit health and reduce MeHg toxicity [10]. 

This study compared the selenoneine and total selenium content of the fish muscle in commercially important fish species in Japan.

## 2. Experimental Section

### 2.1. Materials

The fish samples were obtained at a local market in Tokyo and stored frozen at −40 °C until use. Fillet samples of three individuals were mixed into a single composite sample, and the mean value of three composite samples was determined for the total selenium analysis, except for alfonsino. For the selenium and mercury analysis of alfonsino, 24 fish (average body weight: 1.953 ± 0.584 kg) were used, and various tissues, such as spleen, blood, hepatopancreas, heart, red muscle, white muscle, brain, ovary and testis, were collected from each individual.

### 2.2. Selenium Concentration Measurement

The fish sample (0.1–0.2 g) was digested at 200–220 °C in 1 mL of a 1:2 mixture of nitric acid and perchloric acid. The selenium concentration was determined by hydride generation atomic absorption spectroscopy. For the selenium analysis of the alfonsino tissues, the selenium concentration was determined by a fluorometric assay using 2,3-diamino-naphthalene [8,12].

### 2.3. Mercury Concentration Determination

The total mercury levels were determined by flameless atomic absorption spectrometry at 253.7 nm using an HG-310 mercury analyzer (Hiranuma, Tokyo, Japan) according to the manufacturer’s instructions. The sample material (0.1–0.5 g) was first digested in 2 mL of a 1:2:1 mixture of nitric acid/perchloric acid/sulfuric acid and diluted in water to 25–125 mL.

## 3. Results

The levels of total selenium were determined in the edible portions of various fish and shellfish in Japan (Table 1, Table 2). Alfonsino muscle contained the highest level of selenium (1.40 mg/kg) among the fish muscles examined in this study. High levels of selenium (1.20–1.07 mg/kg) were also found in the salted ovary products of mullet and Pacific herring. In other fish muscles, the selenium levels ranged between 0.12 and 0.77 mg/kg tissue.

**Table 1:** Selenium content in the edible portions of fish and shellfish.

name	species	Japanese name	portion	selenium (mg/kg)
alfonsino	*Beryx splendens*	*kinmedai*	fillet	1.27
Japanese bluefish	*Scombrops boops*	*mutsu*	fillet with skin	0.77
Pacific bluefin tuna	*Thunnus orientalis*	*kuromaguro*	fillet (low-fat)	0.75
			fillet (high-fat)	0.72
albacore	*Thunnus alalunga*	*bin-naga*	fillet	0.75
skipjack	*Euthynnus pelamis*	*katsuo*	fillet with skin	0.62
Japanese flounder	*Paralichthys olivaceus*	*hirame*	fillet with skin ^c^	0.56
			fillet with skin ^w^	0.42
hoki	*Macruronus novaezelandiae*	*hoki*	fillet with skin	0.56
loach	*Misgurnus anguillicaudatus*	*dojou*	whole	0.50
southern black cod	*Dissostichus eleginoides*	*mero*	fillet	0.49
Pacific ocean perch	*Sebastes alutus*	*alaska-menuke*	fillet	0.49
golden-thread	*Nemipterus virgatus*	*itoyoridai*	surimi	0.48
conger pike	*Muraenesox cinereus*	*hamo*	fillet with skin	0.47
yellowtail	*Seriola quinqueradiata*	*buri*	fillet with skin ^w^	0.46
Pacific mackerel	*Scomber japonicus*	*masaba*	fillet with skin	0.40
Pacific herring	*Clupea pallasii*	*nishin*	fillet with skin	0.40
sailfin sandfish	*Arctoscopus japonicus*	*hatahata*	fillet with skin	0.40
gurnard	*Chelidonichthys spinosus*	*houbou*	fillet with skin	0.39
Asian yellowtail	*Seriola lalandi*	*hiramasa*	fillet with skin	0.38
Japanese scallops	*Pecten albicans*	*itayagai*	without shell	0.37
masu salmon	*Oncorhynchus masou*	*sakuramasu*	fillet with skin	0.35
three-line grunt	*Parapristipoma trilineatum*	*isaki*	fillet with skin	0.35
striped jack	*Caranx delicatissimus*	*shima-aji*	fillet with skin ^c^	0.32
Japanese seabass	*Lateolabrax japonicus*	*suzuki*	fillet with skin	0.32
lamprey	*Lethenteron japonicum*	*yatsumeunagi*	fillet with skin	0.32
Pacific halibut	*Hippoglossus stenolepis*	*ohyou*	fillet with skin	0.31
Japanese surf smelt	*Hypomesus pretiosus*	*chika*	fillet with skin	0.31
silver pomfret	*Pampus punctatissimus*	*managatsuo*	fillet with skin	0.31
Japanese common squid	*Todarodes pacificus*	*surumeika*	without viscera	0.30
rainbow trout	*Oncorhynchus mykiss*	*nijimasu*	fillet with skin ^cs^	0.29
			fillet with skin ^cf^	0.26
Japanese parrot fish	*Oplegnathus fasciatus*	*ishidai*	fillet with skin	0.29
coho salmon	*Oncorhynchus kitsch*	*ginzake*	fillet with skin ^c^	0.28
Chinook salmon	*Oncorhynchus tshawytscha*	*masunosuke*	fillet with skin	0.28
Japanese whiting	*Sillago japonica*	*shirogisu*	fillet with skin	0.28
flying fish	*Cypselurus agoo agoo*	*tobiuo*	fillet with skin	0.28
sockeye salmon	*Oncorhynchus nerka*	*benizake*	fillet with skin	0.23
Pacific cod	*Gadus macrocephalus*	*madara*	fillet with skin	0.23
walleye pollock	*Theragra chalcogramma*	*suketoudara*	fillet with skin	0.22
Atlantic mackerel	*Scomber scombrus*	*taiseiyousaba*	fillet with skin	0.23
mullet	*Mugil cephalus*	*mabora*	fillet with skin	0.21
char	*Salvelinus pluvius*	*iwana*	fillet with skin ^c^	0.21
short-neck clam	*Ruditapes philippinarum*	*asari*	without shell	0.21
amago salmon	*Oncorhynchus masou ishikawae*	*amago*	fillet with skin ^c^	0.20
hairtail	*Trichiurus lepturus*	*tachiuo*	fillet with skin	0.19
ocellate puffer	*Takifugu rubripes*	*torafugu*	fillet ^c^	0.17
walleye pollock	*Theragra chalcogramma*	*suketoudara*	surimi	0.16
crucian carp	*Carassius auratus*	*funa*	fillet with skin	0.16
Coho salmon	*Oncorhynchus kitsch*	*ginzake*	fillet with skin ^w^	0.15
Atlantic salmon	*Salmo salar*	*taiseiyousake*	fillet with skin	0.15
purple puffer	*Takifugu porphyreus*	*mafugu*	fillet	0.14
Japanese eel	*Anguilla japonica*	*unagi*	fillet with skin ^c^	0.12

^c^ cultured, ^w^ wild, ^cs^ cultured in sea water, ^cf^ cultured in freshwater.

**Table 2:** Selenium content in the edible portions of fish.

name	species	Japanese name	portion	selenium (mg/kg)
Pacific cod	*Gadus macrocephalus*	*madara*	testis	0.14
Pacific herring	*Clupea pallasii*	*nishin*	ovary	1.07
mullet	*Mugil cephalus*	*mabora*	salted ovary	1.20

**Figure 1:**
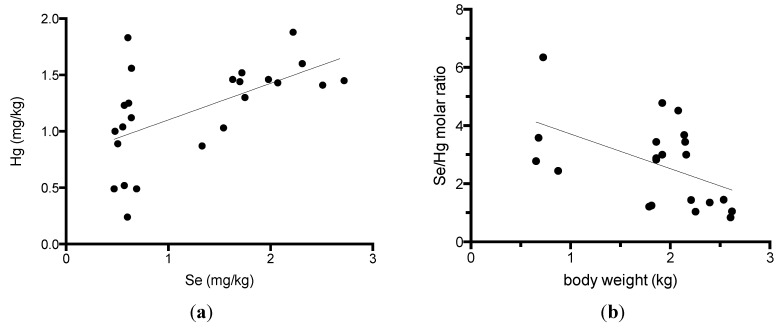
Relationship between the selenium and mercury content in the white and red muscles of alfonsino (*Beryx splendens*). (**a**) Relationship between the selenium and mercury contents; (**b**) Relationship between the body weight and Se/Hg molar ratio.

Because alfonsino muscles were previously shown to contain high levels of selenium and methylmercury [7,11], we compared the selenium and mercury content in the muscles and other tissues in alfonsino (Table 3). The selenium levels were closely correlated with the mercury levels in the white and red muscles in alfonsino (Figure 1a). The correlation coefficients between the selenium and mercury content in the white muscle were calculated to be 0.570 (*p* = 0.004), respectively. All tissues, including spleen, blood, hepatopancreas, heart, red muscle, white muscle, brain, ovary and testis, that were examined in this study contained high levels of selenium, with all being greater than 1 mg/kg, and the highest selenium concentration was determined to be 24.8 mg/kg in the spleen. Se-to-Hg molar ratio in the white muscle of alfonsino was calculated in Fig. 1B. The Se-to-Hg molar ratio ranged from 0.83 to 6.4, and was correlated with the body weight (*r* = −0.504, *p* = 0.020). 

**Table 3:** Distribution of selenium and mercury in the various tissues of alfonsino.

tissue	*n*	Se (mg/kg)	Hg (mg/kg)
spleen	6	24.8 ± 7.18	2.35 ± 0.06
blood	9	17.8 ± 9.32	0.61 ± 0.26
hepatopancreas	6	8.09 ± 2.76	3.92 ± 1.51
heart	6	4.38 ± 1.04	1.24 ± 0.34
red muscle	6	2.71 ± 1.04	1.08 ± 0.20
white muscle	24	1.27 ± 0.77	1.19 ± 0.43
brain	6	1.73 ± 0.22	1.62 ± 0.35
ovary	6	2.43 ± 0.58	0.40 ± 0.12
testis	6	1.10 ± 0.18	0.23 ± 0.12

## 4. Discussion

The selenium levels were determined in tissues of various species of commercially important fish and shellfish in Japan. The highest level of selenium (1.27 mg/kg tissue) was found in the muscle of alfonsino. Other predatory fish species, such as Japanese bluefish (0.77 mg/kg tissue), Pacific bluefin tuna (0.72–0.75 mg/kg tissue), albacore (0.75 mg/kg tissue), skipjack (0.62 mg/kg tissue), Japanese flounder (0.42–0.56 mg/kg tissue) and hoki (0.56 mg/kg tissue), also contained more than 0.5 mg/kg selenium in the muscle. Our previous paper reported that a large proportion of the organic selenium was present as selenoneine in the muscles of these predatory fish species [5,6,7]. However, several fish species, including Japanese conger, Japanese anchovy, chum salmon, Pacific saury, white croaker and marbled sole, contained selenoneine below the level of detection (<0.05 nmol/g tissue) [7]. Selenoproteins, including glutathione peroxidases (GPx) and selenoprotein W, that contain selenium in the selenocysteine form might be present in fish muscles [13,14,15,16,17]. The most important source of selenium in the diets for Japanese is known to be fish [1]. 

Thioredoxin reductase 1 and 2 are known to reduce a variety of small molecules to restore them to forms which can function as antioxidants [18,19]. In addition, selenoneine may be an important component of the redox cycle in animal cells [5,6,7]. GPx and other selenoproteins, the expression of which is induced by selenium intake, are thought to enhance antioxidant activity in animal tissues and cells [13,14,15,16,17]. Selenoneine itself may play a key role as a strong free radical scavenger in a variety of physiological and nutritional processes [5,6,7].

Dietary selenium is postulated to protect against mercury toxicity and to reduce mercury accumulation [8,9,10,11]. Recently, we elucidated that selenoneine is an essential molecule in the MeHg detoxification pathway. Selenoneine was found to accelerate the excretion and demethylation of MeHg by secretory extracellular lysosomal vesicle formation via specific organic cation/carnitine transporter OCTN1 [20]. The dietary intake of selenium by fish consumption might reduce MeHg bioaccumulation and toxicity. The present study indicated that alfonsino white muscle contained the highest levels of selenium and mercury in seafood. The Se-to-Hg molar ratio ranges from 0.83 to 6.4, and decreased in older and larger fish. These findings suggest that mercury may be metabolized in closely related molecular mechanisms as selenium, and both mercury and selenium may be bioaccumulated in marine ecosystems. Our previous paper reported that the Se-to-Hg molar ratio ranges from 1 to 217 in the muscle of various fishes. The animal trials of feeding with both MeHg and sodium selenite showed that the toxicity of MeHg was reduced by selenium intake for a Se-to-Hg molar ratio above 0.2 [19]. Therefore, from the data on total selenium and mercury content, the alfonsino white muscle is thought to represent normal physiological states from the viewpoint of MeHg bioaccumulation and metabolism.

In conclusion, the selenium levels were determined in the edible portion of commercially important fish and shellfish in Japan, ranging between 0.12 and 1.27 mg/kg tissue. In alfonsino, the selenium levels were closely correlated with the mercury levels in the white and red muscles.
